# Simian T-Lymphotropic Virus Diversity among Nonhuman Primates, Cameroon

**DOI:** 10.3201/eid1502.080584

**Published:** 2009-02

**Authors:** David M. Sintasath, Nathan D. Wolfe, Matthew LeBreton, Hongwei Jia, Albert D. Garcia, Joseph Le Doux Diffo, Ubald Tamoufe, Jean K. Carr, Thomas M. Folks, Eitel Mpoudi-Ngole, Donald S. Burke, Walid Heneine, William M. Switzer

**Affiliations:** Johns Hopkins Bloomberg School of Public Health, Baltimore, Maryland, USA (D.M. Sintasath, D.S. Burke); University of California School of Public Health, Los Angeles, California, USA (N.D. Wolfe, U. Tamoufe); Johns Hopkins Cameroon Program, Yaoundé, Cameroon (M. LeBreton, J.L.D. Diffo, U. Tamoufe, E. Mpoudi-Ngole); Centers for Disease Control and Prevention, Atlanta, Georgia, USA (H. Jia, A. D. Garcia, T.M. Folks, W. Heneine, W.M. Switzer); University of Maryland Biotechnology Institute, Baltimore (J.K. Carr); 1 Current affiliation: Stanford University, Stanford, California, USA.; 2 Current affiliation: University of Pittsburgh Graduate School of Public Health, Pittsburgh, Pennsylvania, USA.

**Keywords:** Dried blood spots, retrovirus, simian T-lymphotropic virus, research

## Abstract

Cross-species transmission of retroviruses is common in Cameroon. To determine risk for simian T-cell lymphotropic virus (STLV) transmission from nonhuman primates to hunters, we examined 170 hunter-collected dried blood spots (DBS) from 12 species for STLV. PCR with generic *tax* and group-specific long terminal repeat primers showed that 12 (7%) specimens from 4 nonhuman primate species were infected with STLV. Phylogenetic analyses showed broad diversity of STLV, including novel STLV-1 and STLV-3 sequences and a highly divergent STLV-3 subtype found in *Cercopithecus mona* and *C. nictitans* monkeys. Screening of peripheral blood mononuclear cell DNA from 63 HTLV-seroreactive, PCR-negative hunters did not identify human infections with this divergent STLV-3. Therefore, hunter-collected DBS can effectively capture STLV diversity at the point where pathogen spillover occurs. Broad screening using this relatively easy collection strategy has potential for large-scale monitoring of retrovirus cross-species transmission among highly exposed human populations.

## Abstract 2

Broad virus diversity warrants active monitoring for cross-species transmission and highlights the risk for human disease.

Primate T-lymphotropic viruses (PTLVs) are composed of simian and human T-lymphotropic viruses (STLVs and HTLVs, respectively). To date, only 4 major PTLV groups have been identified. PTLV-1, PTLV-2, and PTLV-3 include human (HTLV-1, HTLV-2, and HTLV-3) and simian (STLV-1, STLV-2, and STLV-3) viruses (*1*–*6*). PTLV-4 consists of only HTLV-4, which was recently reported in a person in Cameroon known to have been exposed to nonhuman primates (NHPs) (*7*). A simian counterpart of this virus has yet to be identified. More recently, a highly divergent STLV-1–like virus from captive macaques (*Macaca arctoides*) has been described (*8*); further analysis suggests a possible new lineage outside the diversity of PTLV-1, provisionally named STLV-5 (*9*).

Both HTLV-1 and HTLV-2 have spread globally and are pathogenic in humans (*10*–*13*). HTLV-1 causes adult T-cell leukemia/lymphoma, HTLV-1–associated myelopathy/tropical spastic paraparesis, and other inflammatory diseases in <5% of those infected (*2*,*11*,*13*). HTLV-2 is less pathogenic than HTLV-1 but has been associated with a neurologic disease similar to HTLV-1–associated myelopathy/tropical spastic paraparesis (*10*,*12*). HTLV-1 and HTLV-2 are known to be transmitted by sexual contact, breast-feeding, and exposure to contaminated blood or blood products through transfusion and injection drug use (*11*–*13*). Less is known about the transmissibility and pathogenicity of HTLV-3 and HTLV-4. Nevertheless, recent full-length sequence analysis of the HTLV-3 (*14*,*15*) and HTLV-4 genomes (W.M. Switzer et al., unpub. data) suggested ancient origins of these viruses and showed functional motifs that affect viral expression and possibly oncogenesis (*14,15*; W.M. Switzer et al., unpub. data).

The recent discovery of HTLV-3 and HTLV-4 demonstrates that the diversity of PTLV is far from understood (*7*). Studies have shown that the diversity of HTLV is directly related to the genetic diversity of the STLVs from which the primary zoonotic infection originated (*5*,*16*). Every HTLV-1 subtype except A is composed of genetically related HTLV-1 and STLV-1 strains from many different primate species, all found geographically near each other. Similarly, PTLV-3s exhibit broad diversity among NHPs in the wild; currently, 3 subtypes have been suggested according to the geographic origin of the strains (*17*): East African STLV-3 subtype A includes STLV-3 (PH969) found in a baboon (*Papio hamadryas*) from Eritrea (*18*) and from captive gelada baboons (*Theropithecus gelada*) (*19*); West and Central African STLV-3 subtype B includes STLV-3 (CTO-604) and STLV-3 (CTO-602) found among mangabeys (*Cercocebus torquatus*) from Cameroon (*20*) and STLV-3 (PPAF3) from baboons (*P. hamadryas papio*) from Senegal (*17*); and Central African STLV-3 subtype C includes divergent strains (Cni217 and Cni227) from *Cercopithecus nictitans* monkeys from Cameroon (*21*). Together, this clustering by geography rather than host species suggests the ease with which STLVs are transmitted among NHPs and possibly to humans (*2*,*3*,*5*,*22*,*23*).

We used a hunter-based field surveillance approach to investigate STLV diversity among primate bushmeat samples collected from 12 NHP species in different locations in Cameroon. We also sampled NHPs in the surrounding region for the STLV source of the HTLV-4–infected individual. In addition, we examined the utility of using dried blood spots (DBS) in the field for surveillance of cross-species transmission of retroviruses.

## Materials and Methods

### Sample Collection and Preparation

**Figure 1:**
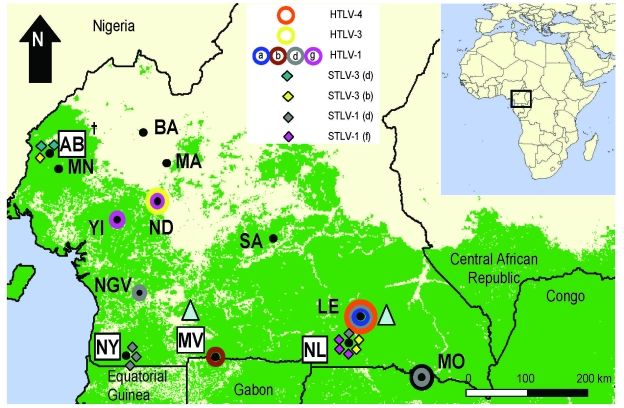
Distribution of primate T-lymphotropic viruses identified in humans and nonhuman primates from rural villages and forests in southern Cameroon. Colored circles and diamonds correspond to human (HTLVs) and simian T-lymphotropic viruses (STLVs) (subtypes), respectively, found at each study site in the current study and reported previously (*7*). Shaded triangles indicate approximate sampling sites where STLV-3–like strains have been reported by others (*9*). The 4 locations where Old World monkey and prosimian species were sampled in this study are boxed in white. AB, Abat; MV, Mvangan; NY, Nyabissan; NL, Ngoila; MN, Manyemen; BA, Bangourain; MA, Massangam; YI, Yingui; ND, Ndikinimeki; NGV, Ngovayan; SA, Sobia; LE, Lomie; MO, Mouloundou.

Before the study began, Institutional Animal Care and Use Committee approvals were obtained. Self-identified hunters from 4 study sites in southern Cameroon volunteered to collect DBS from freshly hunted NHP bushmeat (Figure 1). Hunters were educated about the risks associated with direct contact with NHPs and about appropriate prevention measures. Preliminary identification of hunted species was undertaken by using pictographs of NHPs common in the region (*24*). Confirmation of species was performed by analysis of mitochondrial cytochrome oxidase subunit II and/or glucose-6-phosphate dehydrogenase sequences (*25*,*26*). Over 2 years, a total of 362 DBS from hunted NHPs were collected on Whatman filter paper (Kent, UK), air dried, and stored locally at room temperature in envelopes with dessicant until processed. Nucleic acids were extracted by using the NucliSens nucleic acid isolation kits (bioMérieux, Durham, NC, USA). DNA quality and yield were determined by semiquantitative PCR amplification of the β-actin gene as previously described (*27*,*28*). DNA preparation and PCR assays were performed in different laboratories specifically outfitted for processing and testing of NHP samples only, according to established precautions to prevent contamination. Specimens were coded by using a strategy previously described (*15*).

### PTLV Sequence Detection and Sequence Analysis

DNA samples from NHPs were tested for *tax* sequences by using generic and nested PCR assays capable of detecting viruses from all 4 major PTLV groups (*7*,*19*,*27*). Phylogenetic resolution was achieved by analysis of long terminal repeat (LTR) sequences using PTLV group-specific primers (*7*). PCR amplification of overlapping regions of the 5′ and 3′ STLV-1 LTR (*4*) and partial STLV-3 LTR (*7*,*19*) sequences were performed using primers and conditions reported elsewhere. A PCR-based genome-walking approach (*15*) was used to obtain partial viral genome fragments of a highly divergent PTLV from monkeys Cmo8699AB and Cni7867AB (Table 1). (NHPs are coded as follows: the first letter of the genus is followed by the first 2 letters of the species name, e.g., Cag, *Cercocebus agilis;* Cni, *C. nictitans;* Cmo, *C. mona;* and Lal, *Lophocebus albigena*. The last 2 letters in the code indicate the study site, e.g., AB, Abat; MV, Mvangan.)

**Table 1:** Nucleotide sequences of primer sets used to amplify *tax* and long terminal repeat sequences of simian T-lymphotropic virus–3 (Cmo8699AB and Cni7867AB)*

Region	Primer set	Forward primer and sequence (5′ → 3′)	Reverse primer and sequence (5′ → 3′)	bp
*tax*	Outer	8699TF1 GTACCCTGTCTACGTTTTCGGCGAT	PGTAXR1 GAIGAYTGIASTACYAAAGATGGCTG	779
	Inner	8699TF2 TTACTGGCCACCTGTCCTGAACAC	PGTAXR2 TTIGGGYAIGGICCGGAAATCAT	658
	Outer	P5TAXF3† CCCTCAAGGTCCTCACCCCGCCGC	P5TAXR3 TAACGGCCAGGTCATTGGAGGTGT	244
	Inner	P5TAXF2† AAGTTCCTCCCTCCTTCTTCCATG	P5TAXR1 TGGTAGAGGTATAAGCACACGATGGTG	174
*tax-*LTR	Outer	8699TF6 CATCCGGACCAACTAGGGGCCTTC	PGTATA1+2R1 TCCTGAACYGTCYYYRCGCTTTTATAG	721
	Inner	8699TF7‡ AACAAAAATCCCTACCAAACGCTT	PGTATA1+2R1 TCCTGAACYGTCYYYRCGCTTTTATAG	695
	Inner	8699TF8§ CAGCCCACCCGCGCACCAGTAATT	PGTATA1+2R1 TCCTGAACYGTCYYYRCGCTTTTATAG	589
LTR	Outer	8699LF3 CTCTGACGTCTCTCCCTGCCTTGT	PGPBSR1n ATCCCGGACGAGCCCCCA	612
	Inner	8699LF4 CCGGAAAAAACCTTAAACCACCCA	PGPBSR1n ATCCCGGACGAGCCCCCA	585

*bp, base pair; LTR, long terminal repeat; I, inosine; S, G/C; Y, T/C; R, A/G. Nonhuman primates are coded as follows: the first letter of the genus is followed by the first 2 letters of the species name: Cmo, *Cercopithecus mona*; Cni, *C. nictitans*. The last 2 letters in the code indicate the study site: AB, Abat. †Primers used to screen human peripheral blood mononuclear cell DNA for simian T-lymphotropic virus-3 (Cmo8699AB)–like *tax* sequences. ‡Primer set used for Cni7867AB. §Primer set used for Cmo8699AB.

To screen humans for the divergent STLV-3 subtype, we developed a nested PCR assay based on STLV-3 (Cmo8699AB) *tax* sequences. Similar strategies have been used to screen for the novel HTLV-3 and HTLV-4 viruses in NHP hunters from Cameroon (*1*,*7*). DNA for PCR testing was available from a previous study in which plasma from 63 hunters showed a range of seroreactivity to HTLV antigens by Western blot (WB; Genelabs Diagnostics 2.4 kit [*7*]). WB profiles were HTLV-1–like (n = 2), HTLV-2–like (n = 4), HTLV-positive but untypeable (n = 8), and HTLV-indeterminate (n = 49) (*7*). New STLV-3 (Cmo8699AB)-*tax* specific primers were designed to screen peripheral blood mononuclear cell DNA from all 63 hunters previously negative for sequences by using generic primers that can detect PTLV-1, PTLV-2, PTLV-3, and PTLV-4 (*7*).The assay could reliably detect 10 copies of STLV-3 (Cmo8699AB) *tax* plasmid sequences in a background of human DNA. STLV-3 (Cmo8699AB) *tax* sequences were not amplified from PTLV-1, PTLV-2, PTLV-3, and HTLV-4 cell line DNA or *tax*-containing plasmid DNA or from HTLV nonreactive blood donor DNA samples (data not shown), which shows the high sensitivity and specificity of the assay.

PCR products were purified by using QIAquick PCR or gel purification kits (QIAGEN, Valencia, CA, USA) and were either directly sequenced in both directions on an ABI 3130*xl* sequencer (Applied Biosystems, Foster City, CA, USA) or were sequenced after cloning into a TOPO vector (Invitrogen, Carlsbad, CA, USA). Sequence and phylogenetic analyses were performed according to methods previously described (*15*). Molecular dating of STLV-3 (Cmo8699AB) was based on an alignment of 881-bp *tax* sequences and used previously reported methods (*15*). GenBank accession numbers for the STLV-1 LTR, STLV-3 LTR, STLV-3 (Cmo8699AB) *tax*-LTR, and small *tax* sequences are EU152271–EU152276, EU152277–EU152279, EU152280–EU152281, and EU152282–EU152293, respectively.

## Results

A total of 362 DBS representing 12 NHP and prosimian species were collected (Figure 1), of which 215 (60%) were of adequate quality and quantity for nucleic acid extraction, and 170 (79%) of the 215 yielded adequate amplifiable DNA (Table 2). Blood clots and limited volumes of blood on some DBS accounted for poor DNA yield of some samples.

**Table 2:** Primate T-lymphotropic virus distribution among wild simian and prosimian species, Cameroon*

Taxonomic name (common name)	DBS extracted, no.	β-actin positive, no. (%)	*tax* positive,† no. (%)	STLV-1 LTR positive, no.	STLV-3 LTR positive, no.
Old World monkeys					
*Cercocebus agilis* (agile mangabey)	6	3 (50)	3 (100)	2	1
*C. cephus* (moustached monkey)	41	32 (78)	0	0	0
*C. mona* (mona monkey)	40	36 (90)	1(2.7)	0	1
*C. neglectus* (de Brazza's monkey)	1	1 (100)	0	0	0
*C. nictitans* (spot-nosed monkey)	98	73 (74.5)	7 (9.6)	4	2
*C. pogonias* (crowned monkey)	9	8 (88.8)	0	0	0
*Colobus guereza* (guereza colobus)	3	2 (66.7)	0	0	0
*Lophocebus albigena* (gray-cheeked monkey)	10	9 (90)	1 (11.1)	0	1
Prosimian					
*Arctocebus aureus* (golden angwantibo)	2	1 (50)	0	0	0
*A. calabarensis* (calabar angwantibo)	2	2 (100)	0	0	0
*Galago alleni* (Allen's galago)	1	1 (100)	0	0	0
*Perodicticus potto* (potto)	2	2 (100)	0	0	0
Total	215	170 (79.1)	12 (7.1)	6 (3.5)	5 (2.9)

*DBS, dried blood spots; STLV, simian T-lymphotropic virus; LTR, long terminal repeat. †Samples negative for β-actin sequences were not tested for primate T-lymphotropic virus sequences.

**Figure 2:**
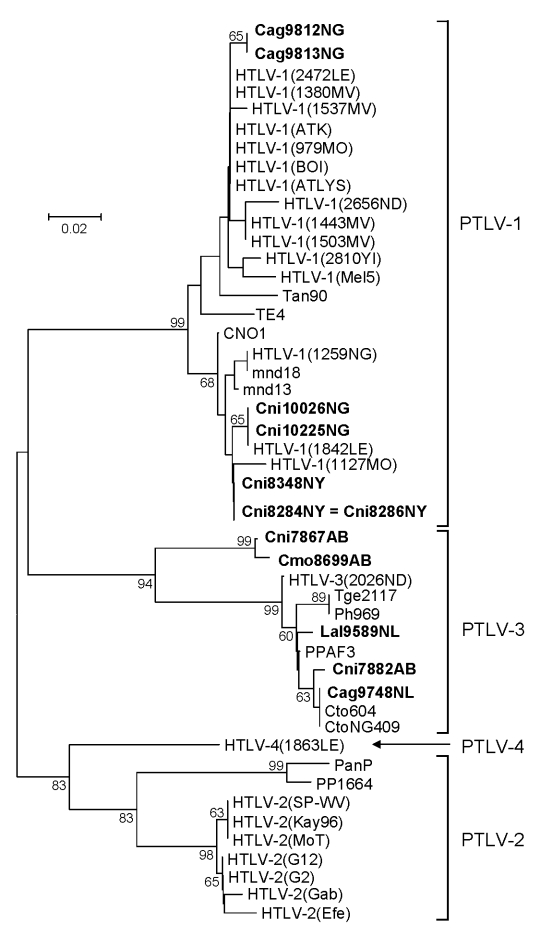
Primate T-lymphotropic virus (PTLV) phylogeny inferred by using 161-bp *tax* sequences. New sequences from nonhuman primates (NHPs) from Cameroon in this study are in **boldface**. Support for the branching order was determined by 1,000 bootstrap replicates; only values >60% are shown. Branch lengths are proportional to the evolutionary distance (scale bar) between the taxa. Nonhuman primates are coded as follows: the first letter of the genus is followed by the first 2 letters of the species name: Cmo, *Cercopithecus mona;* Cni, *Cercopithecus nictitans;* Cto, *Cercocebus torquatus*; Ppa, *Papio papio*; Ph, *Papio hamadryas*; Tge, *Theropithecus gelada.* Cag, *Cercocebus agilis;* Lal, *Lophocebus albigena*; Mnd and msp, *Mandrillus sphinx*; PanP and PP, *Pan paniscus*; Ptr, *Pan troglodytes*; Ggo, *Gorilla gorilla*; Tan, tantalus monkey; Cag, *Cercocebus agilis*; Mar, *Macaca arctoides*; Pha, *Papio hamadryas*; Pan, *Papio anubis*; Bab, baboon; HYB, hybrid baboon (Pha X Pan); Cae, *Chlorocebus aethiops* (AGM, African green monkey); Cpo, *Cercopithecus pogonias*; Cmi, *Cercopithecus mitis*; Cce, *Cercopithecus cephus*; Ang, *Allenopithecus nigroviridis*; Wrc, Western red colobus. The last 2 letters in the code indicate the study site: AB, Abat; MV, Mvangan; NY, Nyabissan; NL, Ngoila; MN, Manyemen; BA, Bangourain; MA, Massangam; YI, Yingui; ND, Ndikinimeki; NG, Ngovayan; SA, Sobia; LE, Lomie; MO, Mouloundou.

Because of the limited amount of DBS material available, we used a PCR assay that detects sequences from all 4 major PTLV groups. We observed a broad range of PTLV diversity over a wide geographic distribution. Of the 170 samples screened, 12 (7%) from 4 NHP species were positive for PTLV *tax* sequences (Table 3). Phylogenetic analysis of the short *tax* sequences from these 12 samples showed that 7 NHPs (2 *Cercocebus agilis* and 5 *C. nictitans* monkeys) were infected with STLV-1 and that 3 (*C. agilis*, *C. nictitans*, and *Lophocebus albigena* monkeys) were infected with STLV-3 (Figure 2; Table 3). We did not find any evidence of STLV-2, HTLV-4–like STLV, or dual STLV-1 and STLV-3 infections as have been found in *C. agilis* monkeys in other studies (*25*).

**Table 3:** Primate T-lymphotropic virus diversity and geographic distribution among wild nonhuman primates, Cameroon*

No.	Code	Species (common name)	Site	Province	PTLV (subtype)
1	Cag9812NL	*Cercopithecus agilis* (agile mangabey)	Ngoila	East	STLV-1 (f)
2	Cag9813NL	*C. agilis*	Ngoila	East	STLV-1 (f)
3	Cag9748NL	*C. agilis*	Ngoila	East	STLV-3 (b)
4	Cmo8699AB	*C. mona (*mona monkey)	Abat	Southwest	STLV-3 (d)
5	Cni10026NL	*C. nictitans* (spot-nosed monkey)	Ngoila	East	STLV-1†
6	Cni10225NL	*C. nictitans*	Ngoila	East	STLV-1 (d)
7	Cni8284NY	*C. nictitans*	Nyabissan	South	STLV-1 (d)
8	Cni8286NY	*C. nictitans*	Nyabissan	South	STLV-1 (d)
9	Cni8348NY	*C. nictitans*	Nyabissan	South	STLV-1 (d)
10	Cni7882AB	*C. nictitans*	Abat	Southwest	STLV-3 (b)
11	Cni7867AB	*C. nictitans*	Abat	Southwest	STLV-3 (d)
12	Lal9589NL	*Lophocebus albigena* (gray-cheeked monkey)	Ngoila	East	STLV-3 (b)

*PTLV, primate T-lymphotropic virus; STLV, simian T-lymphotropic virus. Nonhuman primates are coded as follows: the first letter of the genus is followed by the first 2 letters of the species name: Cag, *C. agilis*; Cmo, *C. mona*; Cni, *C. nictitans*.; Lal, *L. albigena*. The last 2 letters in the code indicate the study site: AB, Abat; NL, Ngoila; NY, Nyabissan. †Subtype not determined.

**Figure 3:**
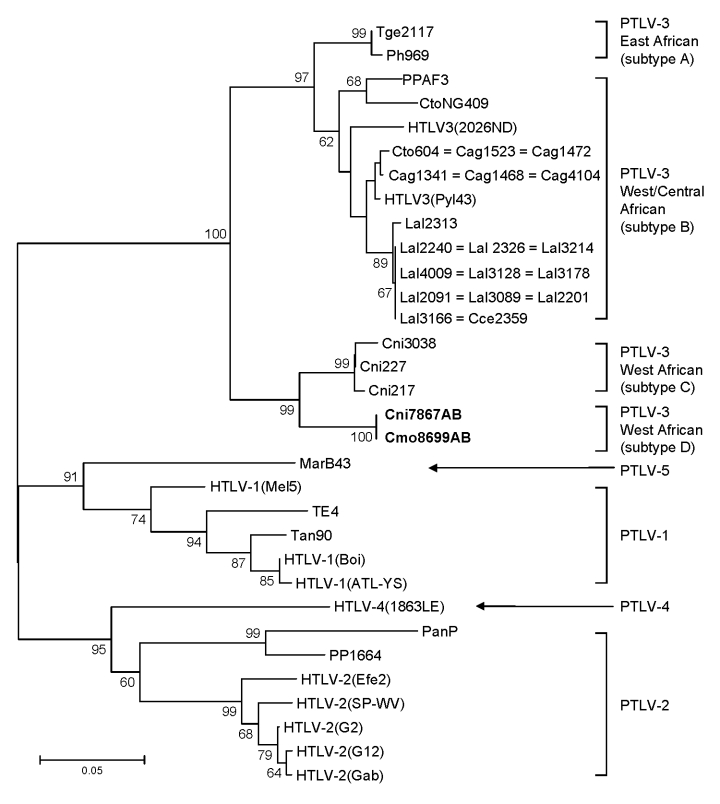
Identification of a novel primate T-lymphotropic virus (PTLV)–3 subtype by phylogenetic inference of 202-bp *tax* sequences with PTLV prototypes and partial sequences from 3 *Cercopithecus nictitans* (Cni217, Cni227, and Cni3038) reported elsewhere (*9*,*21*) and those identified in the current study (in **boldface**). GenBank accession numbers for the previously reported partial simian T-lymphotropic virus (STLV)–3 *tax* sequences included in this analysis are AY039033, AF412120, and AM746647–AM746673). Support for the branching order was determined by 1,000 bootstrap replicates; only values >60% are shown. Branch lengths are proportional to the evolutionary distance (scale bar) between the taxa. See Figure 2 legend for abbreviations.

Samples Cmo8699AB and Cni7867AB, each collected near the same village but from 2 different NHP species, contained nearly identical STLV sequences with highest nucleotide identity to viruses in the PTLV-3 group, but they exhibited high divergence in this small region of *tax* (Figure 2; Table 4). BLAST analysis (www.ncbi.nlm.nih.gov/blast/Blast.cgi) of these divergent *tax* sequences identified sequence similarity (≈92%–93%) to short STLV-3–like *tax* sequences (≈219 bp) from 4 *C. nictitans* monkeys from southern Cameroon (Cni217, Cni227, Cni3034, and Cni3038; GenBank accession nos. AY039033, AF412120, AM746663, and AM746660, respectively) (Table 4) (*9*,*21*). However, further phylogenetic analysis of STLV-3 (Cmo8699AB) and STLV-3 (Cni7867AB), including the small *tax* sequences from 3 of the 4 *C. nictitans* monkeys (Cni3034 was omitted because it had a shorter but identical *tax* sequence to Cni3038) and from other STLV-3–infected species (*L. albigena*, *C. agilis*, and *C. cephus*) from the same region (*9*,*21*), showed that our viruses clustered tightly with high bootstrap support (99%) as a distinct monophyletic subtype of STLV-3 (Figure 3). Because nucleotide divergence is generally <3% within viral subtypes and up to 15% between viral subtypes in the *tax* region (*7*), the 7% divergence seen in the *tax* sequences of STLV-3 (Cmo8699AB) and STLV-3 (Cni7867AB), along with the clustering of these viruses outside the diversity of other STLV-3–like viruses (*9*,*21*), together suggested the identification of a new and highly divergent PTLV-3 subtype (Figure 3; Table 4).

**Table 4:** High genetic diversity of novel STLV-3 (subtype D) *tax* sequences compared to prototypical PTLV-3s* www.cdc.gov/EID/content/15/2/175-T4.htm

Nonhuman primate	Subtype D			Subtype C					Subtype B					Subtype A	
	Cmo 8699AB†	Cni 7867AB†		Cni 217‡	Cni 227‡	Cni 3034§	Cni 3038¶		2026 ND	Cto604	CtoNG 409	PPAF3		Ph969	Tge 2117
Cmo8699AB	**–**	99.9		**92.7**	**93.2**	**93.5**	**93.1**		**82.7**	**83.4**	**83.5**	**83.5**		**84.5**	**84.2**
Cni7867AB		–		**92.7**	**93.2**	**93.5**	**93.1**		**82.7**	**83.4**	**83.5**	**83.5**		**84.5**	**84.2**
Cni217				–	99.5	98.2	98.5		**84.5**	**86.3**	**88.1**	**86.8**		**88.6**	**88.1**
Cni227					–	98.8	99.1		**84.9**	**86.8**	**87.7**	**87.2**		**89.0**	**88.6**
Cni3034						–	100.0		**82.2**	**82.4**	**82.8**	**83.6**		**83.9**	**83.7**
Cni3038							–		**82.5**	**82.7**	**83.1**	**83.7**		**84.1**	**83.9**
2026ND									–	91.6	93.0	94.1		**87.0**	**90.4**
Cto604										–	92.4	92.5		**87.5**	**92.0**
CtoNG409											**–**	94.2		**86.8**	**90.7**
PPAF3												**–**		**88.5**	**90.8**
Ph969														**–**	95.8
Tge2117															**–**

*STLVs, simian T-lymphotropic viruses; PTLVs, primate T-lymphotropic viruses. **Boldface** indicates intersubgroup identities; shading indicates intrasubgroup identities. Nonhuman primates are coded as follows: the first letter of the genus is followed by the first 2 letters of the species name: Cmo, *Cercopithecus mona;* Cni, *C. nictitans;* Cto, *Cerococebus torquatus*; Ppa, *Papio papio*; Ph, *Papio hamadryas*; Tge, *Teropithecus gelada.* The last 2 letters in the code indicate the study site: AB, Abat; ND, Ndikinimeki. †Partial *tax* sequence (1015 bp). ‡Partial *tax* sequence (219 bp). §Partial *tax* sequence (170 bp). ¶Partial *tax* sequence (202 bp).

### Phylogenetic Resolution of a Novel PTLV-3 Subtype

**Figure 4:**
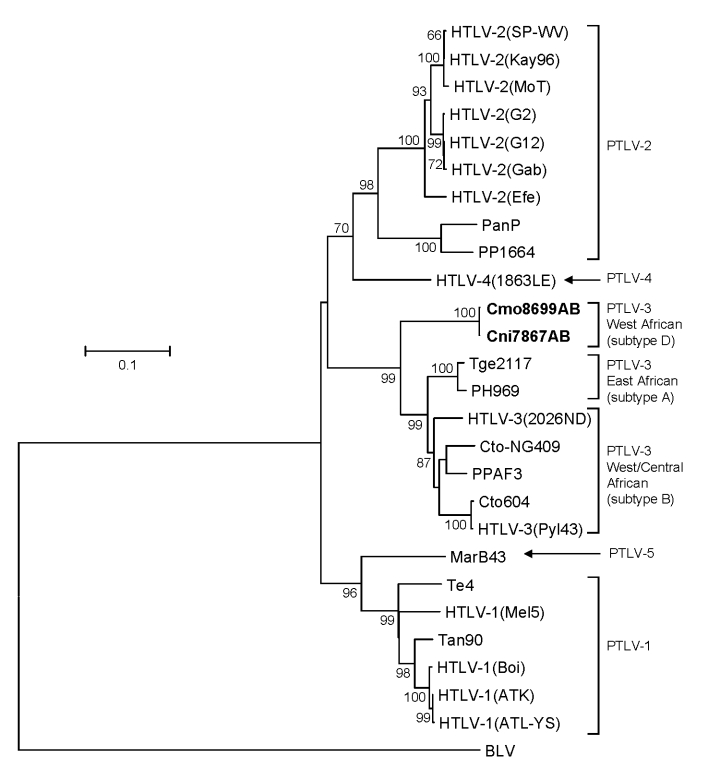
Identification of a novel primate T-lymphotropic virus (PTLV) subtype by phylogenetic inference of 881-bp *tax* sequences from prototypical PTLVs. Bovine leukemia virus (BLV) *tax* sequences were used as an outgroup in the maximum-likelihood analysis. New sequences from this study are in **boldface**. Support for the branching order was determined by 1,000 bootstrap replicates; only values >60% are shown. Branch lengths are proportional to the evolutionary distance (scale bar) between the taxa. See Figure 2 legend for abbreviations.

The identification of highly divergent STLV-3–like sequences in Cmo8699AB and Cni7867AB was investigated further by additional analyses of a larger *tax* sequence (1,015 bp). Both *tax* sequences were nearly identical (99.9%) despite nucleic acid extraction, PCR amplification, and sequencing for both animals all being performed on different days. Analysis of mitochondrial DNA sequences also confirmed the *Cercopithecus* species of each monkey and the absence of admixtures of specimens from different NHP species. STLV-3 (Cmo8699AB) *tax* sequences share 72%–74% nucleotide identity with PTLV-1, PTLV-2, and PTLV-4, but they have the highest nucleotide identity to the PTLV-3 group (82%–84%) in this highly conserved region where intragroup sequence identity is typically >90%. Phylogenetic analysis of 881-bp *tax* sequences (Figure 4) from these 2 monkeys with other PTLVs, using bovine leukemia virus as an outgroup, inferred a new lineage with high bootstrap support (99%) from the diversity of other PTLV-3 subtypes (larger *tax* sequences representing PTLV-3 subtype C were not available for inclusion in this analysis), which suggests a long, independent evolution of this divergent virus.

**Figure 5:**
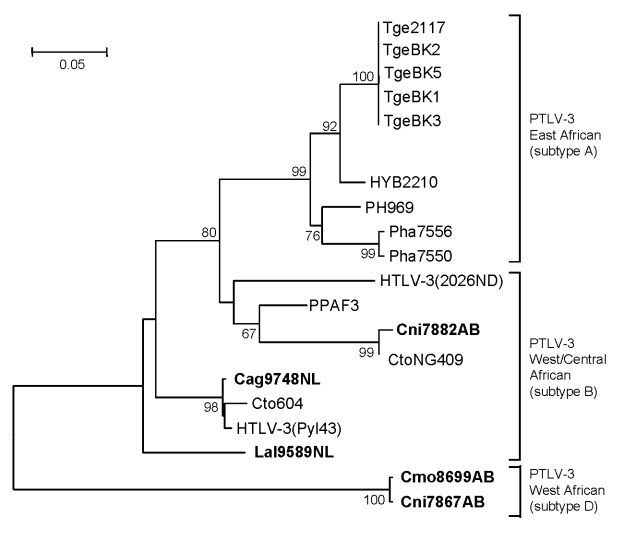
Identification of a novel primate T-lymphotropic virus (PTLV)–3 subtype by phylogenetic analysis of 275-bp long terminal repeat (LTR) sequences. LTR sequences for PTLV-3 subtype C were not available for this analysis. New sequences from this study are in **boldface**. Support for the branching order was determined by 1,000 bootstrap replicates; only values >60% are shown. Branch lengths are proportional to the evolutionary distance (scale bar) between the taxa. BLV, bovine leukemia virus. See Figure 2 legend for additional abbreviations.

Similar PTLV-3 tree topologies were obtained by analysis of 275-bp LTR sequences (Figure 5) in which STLV-3 (Cmo8699AB) and STLV-3 (Cni7867AB) had only 70%–74% identity to LTRs from other members of the PTLV-3 group that share >84% nucleotide identity between subtypes A and B (data not shown). LTR sequences from other STLV-3-infected *C. agilis* and *C. nictitans* monkeys from Cameroon reported elsewhere were not available from GenBank (*9*,*21*,*25*) and thus were not included in the current phylogenetic analysis. Combined, the phylogenetic analyses of the *tax* (Figures 3, 4) and LTR (Figure 5) sequences show that STLV-3 (Cmo8699AB) and STLV-3 (Cni7867AB) each form a distinct cluster with high bootstrap support from the other known PTLV-3 subtypes. On the basis of nomenclature proposed by others (*17*), our results suggest that these viruses are members of a novel PTLV-3 subtype that we tentatively name as STLV-3 West African subtype D.

### Origin of STLV-3 (Cmo8699AB)

To estimate the divergence times of the most recent common ancestor of STLV-3 (Cmo8699AB), we performed additional molecular analyses. We found that the molecular clock hypothesis was not rejected for the 881-bp alignment of PTLV and bovine leukemia virus *tax* sequences in both PAUP* (http://paup.csit.fsu.edu) and Tree-Puzzle (www.tree-puzzle.de) analyses (p = 0.012 and 0.858, respectively), which is consistent with results obtained recently by others (*29*). Using a molecular clock model and a tree calibration date estimated for the origin of Melanesian HTLV-1 ≈40,000–60,000 years ago (*15*,*19*,*29*,*30*), we inferred the evolutionary rate for PTLV to be 9.17 × 10^–7^ to 1.38 × 10^–6^ substitutions/site/year, which is consistent with rates determined previously both with and without a molecular clock model of evolution (*15*,*17*,*20*,*29*–*31*). The evolutionary rate for STLV-3 (Cmo8699AB) is estimated to be 2.11 × 10^–6^ to 3.16 × 10^–6^, and the most common recent ancestor is inferred to have occurred ≈92,072–138,560 years ago, which suggests an ancient origin and perhaps the identification of one of the oldest viruses in the PTLV-3 group.

### Broad STLV-3 Diversity in Wild NHPs

Sequence analysis of the STLV-3 LTR sequences from Cni7882AB, Cag9748NL, and Lal9589NL showed that all were infected with distinct STLV-3s. LTR sequences (283 bp) from animal Cag9748NL shared the greatest identity (>97%) with those from HTLV-3 (Pyl43) and STLV-3 (Cto604) from a red-capped mangabey from Cameroon (*1*,*20*). The 282-bp LTR sequence from Cni7882AB shared the highest nucleotide identity (99%) to STLV-3 (CtoNG409), a red-capped mangabey from neighboring Nigeria (*31*). The phylogeographic clustering of these sequences supports further the proposed subtype classification of STLV-3 by geographic origin rather than by host species (*17*,*19*,*20*,*25*,*31*). In contrast, the 432-bp LTR sequence from *L. albigena* mangabeys (Lal9589NL) was more divergent; it shared only 10%–16% nucleotide identity with all PTLV-3 LTR sequences. Similar to the phylogenetic relationships inferred with the small *tax* sequences, the LTR sequence from *L. albigena* mangabeys (Lal9589NL) formed a new lineage within the diversity of other PTLV-3 sequences from west-central Africa (Figure 5). Although these results need to be confirmed with additional LTR sequences from this virus and from other STLV-3–infected *L. albigena* mangabeys (*9*), our findings demonstrate a host range and geographic distribution of STLV-3 that is more widespread than previously considered.

### Phylogenetic Analysis of STLV-1 Diversity

**Figure 6:**
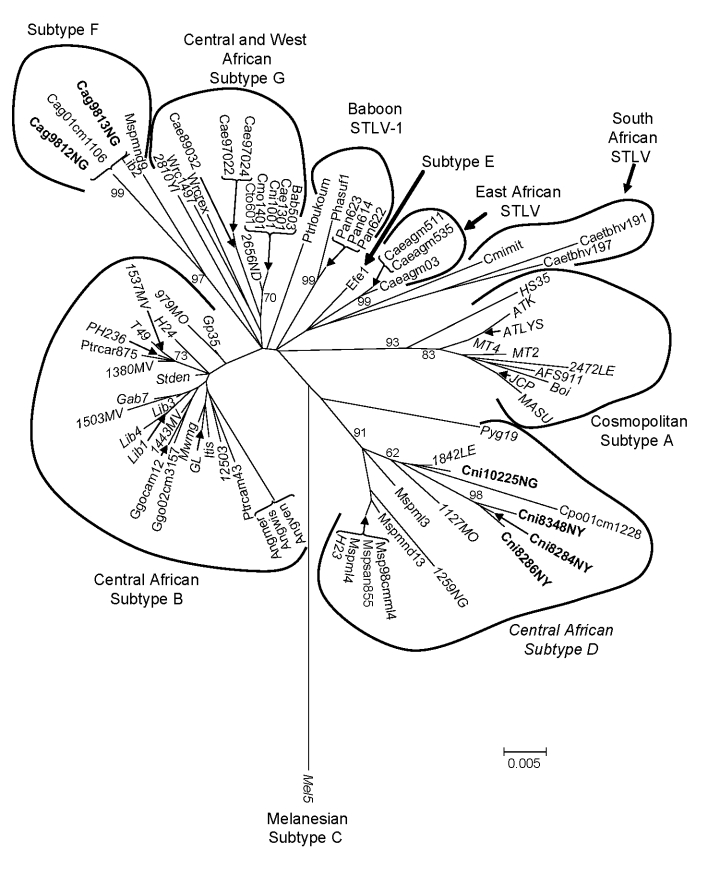
Inferred phylogenetic relationships of primate T-lymphotropic virus (PTLV)–1 long terminal repeat (LTR) sequences by neighbor-joining analysis. Sequences from wild nonhuman primates (NHPs) in Cameroon generated in the current study are in **boldface**. Human T-lymphotropic virus–1 seqences are italicized. Support for the branching order was determined by 1,000 bootstrap replicates; only values >60% are shown. STLV, simian T-lymphotropic virus. See Figure 2 legend for additional abbreviations.

To investigate further the genetic relationships inferred with the small PTLV-1–like *tax* sequences, we obtained LTR sequences for 6 of 7 PTLV-1–positive samples by using established primer-pair combinations (*3*,*4*,*7*). Phylogenetic analysis of these sequences, including those identified from our study of infected NHP hunters in Cameroon (*7*), showed that 4 sequences from *C. nictitans* monkeys all clustered in the central African HTLV-1 subtype D clade, consisting of STLV-1 from *Mandrillus sphinx* and *Cercopithecus pogonias* monkeys and HTLV-1 sequences from Cameroon (Figure 6). The STLV-1 (Cni10225NL) LTR sequence was phylogenetically closest to the HTLV-1 (1842LE) strain from an NHP hunter from Cameroon (*7*) (Figure 6). Similarly, LTR sequences from 2 *C. agilis* (Cag9812NL and Cag9813NL) monkeys clustered within the HTLV-1F clade (Figure 6). Combined, these results support further the primate origin of the HTLV-1D and -1F subtypes. We were unable to amplify STLV-1 LTR sequences from DBS samples from a *C. nictitans* monkey (Cni10026NL) that was positive for STLV-1 *tax* sequences, possibly because of low viral load in this animal, lower sensitivity of the LTR primers, or genetic variances at the LTR primer binding sites. The absence of STLV-1 LTR sequences in this monkey is not likely to have resulted from infection with an STLV-1/STLV-3 recombinant after dual infection of animal Cni10026NL with both viruses because samples from this animal were repeatedly negative for STLV-3 *tax* and LTR sequences.

### Absence of Novel STLV-3 Subtype Sequences in NHP Hunters

Given the prevalence of the STLV-3 subtype D virus in at least 2 monkey species in Cameroon, we investigated whether this new subtype was also present among NHP hunters in Cameroon. Peripheral blood mononuclear cell DNA samples were available from a previous study of 63 NHP hunters who had a wide range of WB seroreactivity to HTLV (*7*). HTLV sequences were not previously detected in the DNA of these persons when either generic or group-specific primers were used (*7*). All 63 NHP hunters were also negative for STLV-3 (Cmo8699AB) *tax*-specific sequences, which suggests the absence of this virus in this subset of persons with broad WB seroreactivity to HTLV.

## Discussion

Widespread exposure to a broad range of NHP body fluids and tissues encountered during hunting, butchering, or keeping primates as pets has been implicated in the emergence of 3 different retrovirus genera: HIV, HTLV, and, more recently, simian foamy virus (*2*–*5*,*7*,*16*,*28*,*32*). Although little is known about the public health implications of simian foamy virus infection, the social, medical, political, and economic consequences of HIV and HTLV global spread and pathogenicity after cross-species transmission are enormous. The recent discovery of HTLV-3 and HTLV-4 in NHP hunters from Cameroon doubles the number of known deltaretroviruses in humans (*7*). This same study also identified novel STLV-1–like infections in NHP hunters (*7*). These discoveries demonstrate that the diversity of PTLV is far from understood and that zoonotic infection with STLV continues in persons exposed to NHPs (*7*). Thus, understanding the diversity, prevalence, and geographic range of STLV infection in areas where frequent contact with wild NHPs is common provides useful information about the origin and emergence of HTLV and the risks for exposure to these and possibly other simian viruses.

We demonstrated that monkeys from 3 distant locations in the rain forests of southern Cameroon are infected with a broad range of highly diverse STLV. Our detection of a 7% prevalence of STLV infection among hunted wild monkeys is comparable to the 8%–11% seroreactivity to PTLV recently found in monkey and ape samples collected mostly at urban bushmeat markets in Cameroon (*9*,*25*). Through analysis of LTR and larger *tax* sequences from *C. mona* and *C. nictitans* monkeys in our study, we have identified new divergent STLV-3–like strains that form a unique PTLV-3 clade that we designated subtype D. Altogether, these results extend further the range of PTLV diversity and suggest a founder effect for PTLV evolutionary radiation in this region where most PTLV groups have been identified.

Given the propensity of STLV to cross species boundaries, the increased frequency of hunting and demand for primate bushmeat in Africa, and the apparent broad diversity of STLV subtypes in Cameroon (*9*,*21*), it is tempting to speculate that human infection with this unique STLV-3 subtype will or may have already occurred. However, PCR testing of DNA samples from Cameroon NHP hunters with broad HTLV WB patterns showed no evidence of STLV-3 (Cmo8699AB)–like infections. Possible explanations for this negative finding include the testing of only a limited number of available samples, an unknown sensitivity for serologic detection of this virus with assays used in our study (*7*), an unknown prevalence and host range of this virus in NHPs, and other factors such as low transmissibility to humans. Nevertheless, the discovery of this novel PTLV-3 subtype in 2 monkey species and an apparent ancient origin of this lineage suggest a possible wider distribution of this variant. Therefore, the ease with which STLVs can cross species barriers and potentially be transmitted during NHP-hunting practices warrants increased surveillance for human infection with this divergent subtype. A similar strategy involving intensified screening of NHP hunters was successful in the discovery of HTLV-3 (*1*,*7*) and HTLV-4 (*7*).

Finding a broad range of STLVs in simian DBS indicates that persons exposed to NHPs from Cameroon are at increased risk for infection with highly diverse STLV. Indeed, phylogenetic analysis of PTLV-1 LTR sequences shows that the new STLV-1 from *C. nictitans* monkeys identified in the current study is most similar to HTLV-1 from Cameroon NHP hunters (*7*). Similarly, the clustering of STLV-1 from *C. agilis* monkeys with LTR sequences obtained from a person from Liberia provides additional support for the primate origin of the HTLV-1F clade (*33*). Combined, these findings further support the hypothesis of active cross-species transmission of STLV to humans in this region (*7*).

Moreover, we show that use of DBS collected in the field in collaboration with hunters provides a good tool for surveillance of emerging retroviral infections at the NHP-hunter interface. Convenient and cost-effective, this collection strategy provides a unique opportunity to examine zoonotic transmission at the point where pathogen spillover occurs. In conjunction with longitudinal sampling of hunters, these collections have the potential to enable simultaneous documentation of both sides of a cross-species transmission event.

In summary, we found broad diversity of STLV in NHPs from Cameroon and identified a novel STLV-3 subtype. These results provide increasing evidence that the diversity and geographic distribution of PTLVs are much greater than previously thought. Bushmeat hunting, an ancient and common practice in many parts of Africa, is an ideal interface for cross-species transmission of retroviruses between NHPs and humans. Contact with body fluids and blood during hunting and butchering of NHP bushmeat exposes humans to a plethora of simian retroviruses, as demonstrated here and elsewhere (*7*,*23*,*25*,*32*,*34*,*35*), and increases the likelihood of emerging diseases in humans. To predict and possibly prevent the next retrovirus pandemic, expanded surveillance is needed for these and other retroviruses in their natural host reservoirs and in persons exposed to NHPs (*7*,*36*,*37*).
